# Relating relapse and T2 lesion changes to disability progression in multiple sclerosis: a systematic literature review and regression analysis

**DOI:** 10.1186/1471-2377-13-180

**Published:** 2013-11-19

**Authors:** Kyle Fahrbach, Rachel Huelin, Amber L Martin, Edward Kim, Homa B Dastani, Stephen Rao, Manoj Malhotra

**Affiliations:** 1 Evidera, 430 Bedford Street, Suite 300, Lexington, MA 02420, USA; 2 Novartis Pharmaceuticals Corporation, One Health Plaza, USEH 135-356, East Hanover, NJ 07936-1080, USA; 3 Bristol-Myers Squibb Company, Route 206 & Province Line Road, Princeton, NJ 08543, USA; 4 Cleveland Neurological Clinic, Cleveland, OH 44195, USA; 5 Questcor Pharmaceuticals, 26118 Research Road, Hayward, CA 94545, USA

**Keywords:** Relapsing-remitting multiple sclerosis, Secondary progressive multiple sclerosis, Relapse, Disability progression, T2 lesions

## Abstract

**Background:**

In the treatment of multiple sclerosis (MS), the most important therapeutic aim of disease-modifying treatments (DMTs) is to prevent or postpone long-term disability. Given the typically slow progression observed in the majority of relapsing-remitting MS (RRMS) patients, the primary endpoint for most randomized clinical trials (RCTs) is a reduction in relapse rate. It is widely assumed that reducing relapse rate will slow disability progression. Similarly, MRI studies suggest that reducing T2 lesions will be associated with slowing long-term disability in MS. The objective of this study was to evaluate the relationship between treatment effects on relapse rates and active T2 lesions to differences in disease progression (as measured by the Expanded Disability Status Scale [EDSS]) in trials evaluating patients with clinically isolated syndrome (CIS), RRMS, and secondary progressive MS (SPMS).

**Methods:**

A systematic literature review was conducted in Medline, Embase, CENTRAL, and PsycINFO to identify randomized trials published in English from January 1, 1993-June 3, 2013 evaluating DMTs in adult MS patients using keywords for CIS, RRMS, and SPMS combined with keywords for relapse and recurrence. Eligible studies were required to report outcomes of relapse and T2 lesion changes or disease progression in CIS, RRMS, or SPMS patients receiving DMTs and have a follow-up duration of at least 22 months. Ultimately, 40 studies satisfied these criteria for inclusion. Regression analyses were conducted on RCTs to relate differences between the effect of treatments on relapse rates and on active T2 lesions to differences between the effects of treatments on disease progression (as measured by EDSS).

**Results:**

Regression analysis determined there is a substantive clinically and statistically significant association between concurrent treatment effects in relapse rate and EDSS; *p* < 0.01. Lower treatment effects were associated with higher relative rates of disease progression. Significant associations between T2 lesion measures and EDSS measures also were found (*p* < 0.05), with some suggestion that the strength of the association may differ for older versus newer DMTs.

**Conclusions:**

Treatment differences in relapse reduction and T2 lesions are positively related to differences in disease progression over the first two years of treatment.

## Background

In the treatment of multiple sclerosis (MS), the most important therapeutic aim of disease-modifying treatments (DMTs) is to prevent or postpone long-term disability, typically defined by worsening on the Expanded Disability Status Scale (EDSS) [1]. Given the normally slow progression observed in the majority of relapsing-remitting MS patients (RRMS) [2], the primary endpoint for most randomized clinical trials (RCTs) is a reduction in relapse rate. It is widely assumed that reducing relapse rate will slow disability progression [3]. Similarly, magnetic resonance imaging (MRI) studies suggest that reducing T2 lesions [4], another short-term outcome in RCTs, will be associated with slowing long-term disability in MS [5].

Sormani and colleagues performed a quantitative meta-analysis of the predictive power of annualized relapse rates and new/enlarged T2 lesions on EDSS progression across a wide range of RCTs for RRMS [6]. Their results indicated that the therapeutic benefit of the drugs, defined by relapse rates and T2 lesions, generally correlated with disability progression. The Sormani et al. study, however, included an intercept in their regression analyses. Inclusion of a non-zero intercept implies that two treatments that do not differ in affecting the predictor (i.e., two treatments that have the same relapse rate) will differ in affecting the outcome (i.e., they will lead to different amounts of EDSS progression). While it is conceptually possible for some pairs of treatments, it would have to hold for all pairs of treatments for the regression to be generalizable. Furthermore, their analysis was limited to RCTs directed at RRMS patients, thus restricting the conclusions that can be drawn regarding the predictive relationship between relapse rates and disability progression in other forms of MS.

In this quantitative meta-analysis, we have extended the Sormani et al. analyses by including data from DMTs involving secondary progressive MS (SPMS) patients. In addition, we have expanded the MRI predictors to include T2 lesion volume, a potentially better predictor of disability progression than new and enlarging T2 lesions. Finally, our statistical approach, which excludes the use of an intercept in the regression analyses, should provide a more meaningful prediction of the relative risk of EDSS progression from treatment differences in the surrogate endpoints of interest (relapse rate and T2 lesions).

## Methods

To identify and retrieve all potentially relevant trials assessing treatment to delay or avoid relapse and disability progression in patients with RRMS, SPMS, and clinically isolated syndrome (CIS), we conducted literature searches in Medline (via PubMed), Embase, Cochrane Central Register of Clinical Trials (CENTRAL), and PsycINFO. The following algorithm was used in PubMed and analogous searches were developed for the remaining databases:

1. “Multiple Sclerosis, Relapsing-Remitting”[Mesh] OR “Relapsing-Remitting Multiple Sclerosis” OR “secondary progressive multiple sclerosis” OR (“Multiple Sclerosis”[Mesh] AND “secondary progressive”[TIAB]) OR “clinically isolated syndrome” OR “early MS” OR “early multiple sclerosis” OR “clinically definite MS” OR “clinically definite multiple sclerosis”.

2. relapse OR relapses OR relapsed OR recurrence.

3. #1 AND #2.

Limits: Humans, English, clinical trial, Not reviews, editorials, comments or case reports, and published between January 1, 1993 and June 3, 2013, with an abstract.

Eligible studies included RCTs of at least 22 months’ duration assessing treatment of MS with DMTs that reported both relapse and disability progression. The Cochrane Library was also searched for recent systematic reviews of the subject, which could be used as a source of further references. A manual check of reference lists from all included studies and relevant reviews/meta-analyses was performed to supplement the above searches and ensure a comprehensive review. Conference abstracts and unpublished literature were not included.

### Study selection

The full text articles of accepted abstracts that passed the initial screening underwent review by investigators trained in systematic review procedures and each excluded study required the consensus of an independent investigator. Abstracts were included when all of the following were true: the study was an RCT evaluating a minimum of 20 adults with CIS, RRMS, or SPMS with at least 22 months of follow-up in which relapse rate or MRI lesion data and disability progression related to treatment with DMTs (both approved and non-approved) were reported, and the study was published within the search period (January 1, 1993-June 3, 2013) for this review.

### Data extraction

Data were collected into an electronic database developed specifically for this review by a single investigator and independently verified by a second investigator. Discrepancies in data extraction were reviewed by the two investigators, and when necessary, any unresolved discrepancies were resolved by a third investigator. The endpoints sought for data capture included annualized relapse rate (ARR), mean change in EDSS, the proportion of patients with disability progression, as well as counts and volume changes for T2 and gadolinium lesions. Definitions for relapse and disease progression were also extracted as defined by authors to ensure similar methods were used in determining the presence of these endpoints across studies.

### Statistical methods

Regression analyses were conducted on RCTs to relate differences between the effect of treatments on relapse rates and on active T2 lesions, to differences between the effects of treatments on disability progression, as measured by EDSS.

The statistical methods used were similar to methods outlined in meta-analyses conducted by Johnson et al. [7] and Sormani et al. [6]. Specifically, weighted least-squares regressions (in which the weight is the total sample size of the two arms being compared) were conducted for combinations of predictor and outcome listed below. However, the analyses were not identical; unlike Sormani et al. [6], we did not weight by duration of follow-up and did not include an intercept in the regression analyses. As noted in the introduction, inclusion of a non-zero intercept implies that two treatments with similar relapse rates could lead to a different degree of EDSS progression. While this is conceptually possible for some pairs of treatments, it would have to hold for all pairs of treatments for the regression to be generalizable.

Regressions were conducted for the following combinations of strata and predictor/outcome pairings:

#### ***Stratifications***

• RRMS, limited to studies in which both arms have approved DMTs (e.g., excluding studies with alemtuzumab, statin-add-on, azathioprine-add-on, cladribine tablets, MBP8298).

• All RRMS, all DMTs.

• SPMS/SPMS mixed.

Overall analyses across all disease courses (including CIS) were also planned; however, given the important clinical differences between disease stages with regard to baseline rates of relapse, it was judged more appropriate to analyze RRMS and SPMS studies independently. CIS studies were not investigated independently, given the sparseness of data available.

Regressions were conducted for the following combinations of strata and predictor/outcome pairings:

#### ***Predictors/outcomes***

• Ratio of ARR as a predictor of the relative risk of EDSS progression (i.e., the ratio of the proportions of patients with a predefined threshold of EDSS increase).

• Ratio in number of new (or new/enlarged) T2 lesions as a predictor of the relative risk of EDSS progression.

• Ratio of follow-up median T2 lesion volumes as a predictor of the relative risk of EDSS progression.

One other analysis was planned *a priori*; however, it was not feasible due to sparseness of data:

• Ratio of gadolinium (GD) lesion volume as a predictor of the relative risk of EDSS progression (i.e., the ratio of the proportions of patients with a predefined threshold of EDSS increase).

In cases where the relative risk of EDSS progression was not available or calculable, the hazard ratio for time-to-progression was used if it was available.

All calculations were performed using SAS^®^ software version 9.2 and SPSS^®^ software version 15.0.

## Results

**Figure 1:**
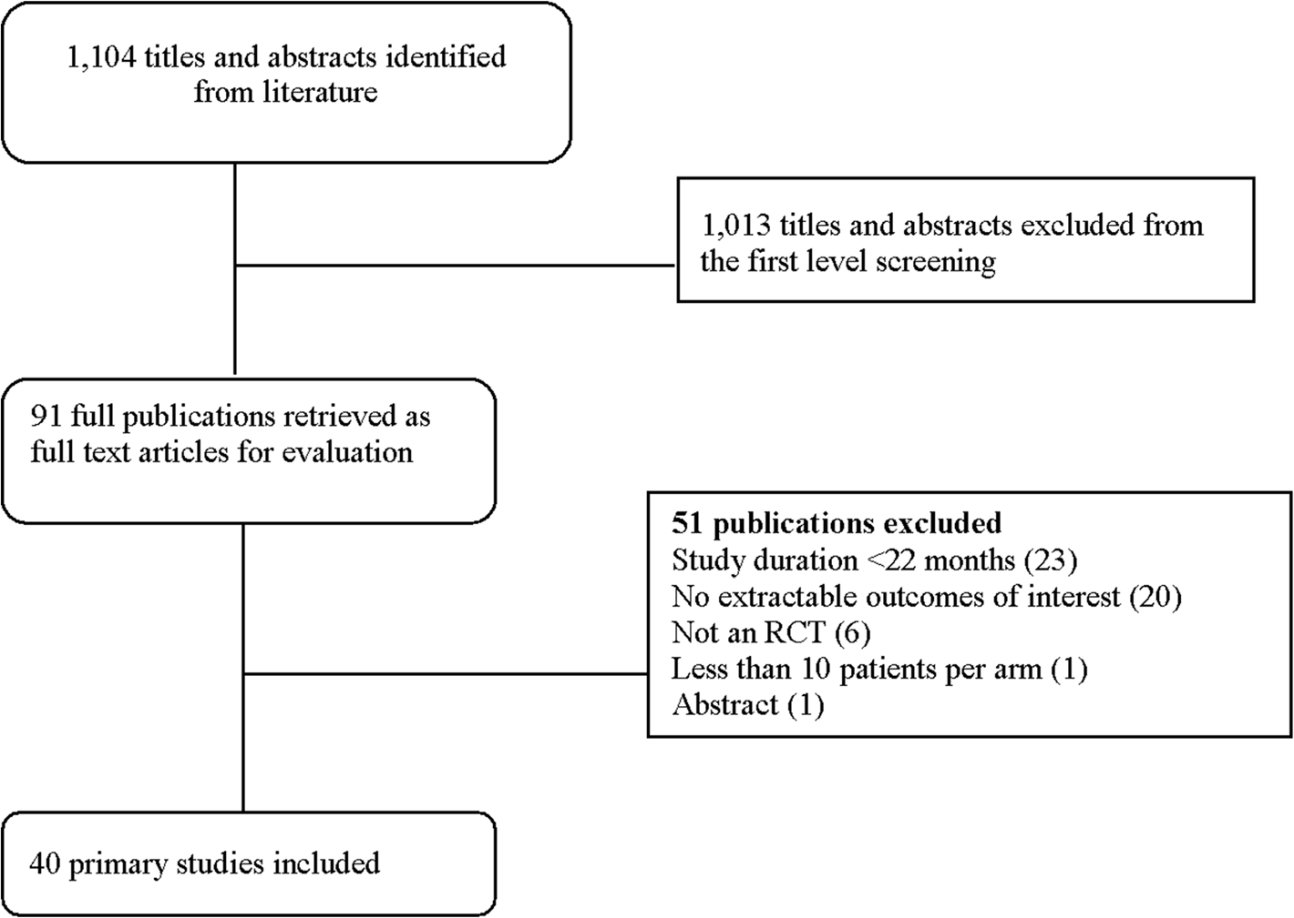
Study attrition.

A total of 1,104 unique citations were identified for review at the abstract level. Of these, 91 abstracts were selected for further review as full-text articles, and 1,013 were excluded. The primary reason for exclusion following full text review was study duration less than 22 months (n = 23), followed by no extractable outcomes of interest (n = 20). Figure 1 presents the study attrition of the literature review. Forty primary articles [8-47] that examined relapse rate and disability progression related to treatment with DMTs in adults with CIS, RRMS, or SPMS with at least 22 months of follow-up were identified for inclusion after full text review.

Of the 40 identified studies, the majority were on RRMS populations (30), followed by SPMS (7). There were only two CIS studies that met our inclusion criteria, as well as one trial evaluating a mixed RRMS and SPMS population.

**Figure 2:**
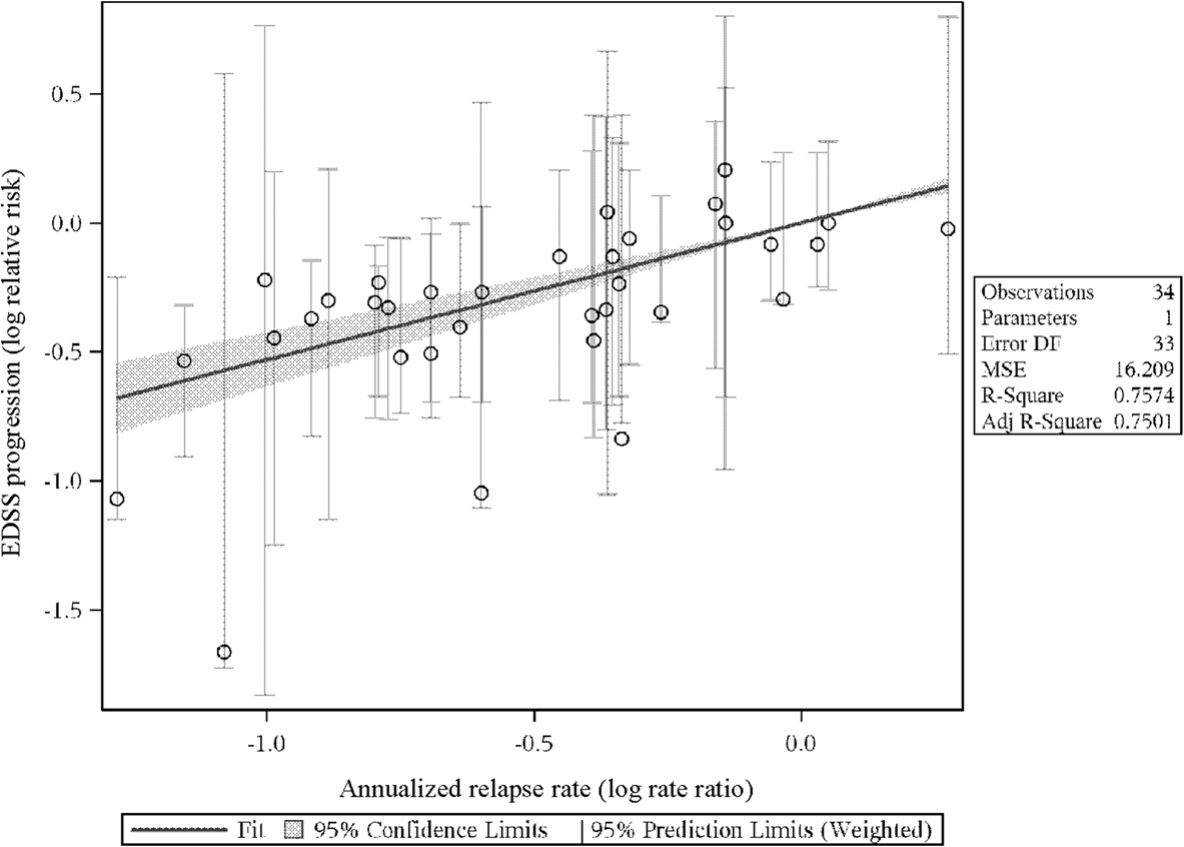
**Predicting log-relative risk of EDSS from log-rate ratio of relapse rate: RRMS, all DMTs.** EDSS: Expanded Disability Status Scale; Log RR.

**Figure 3:**
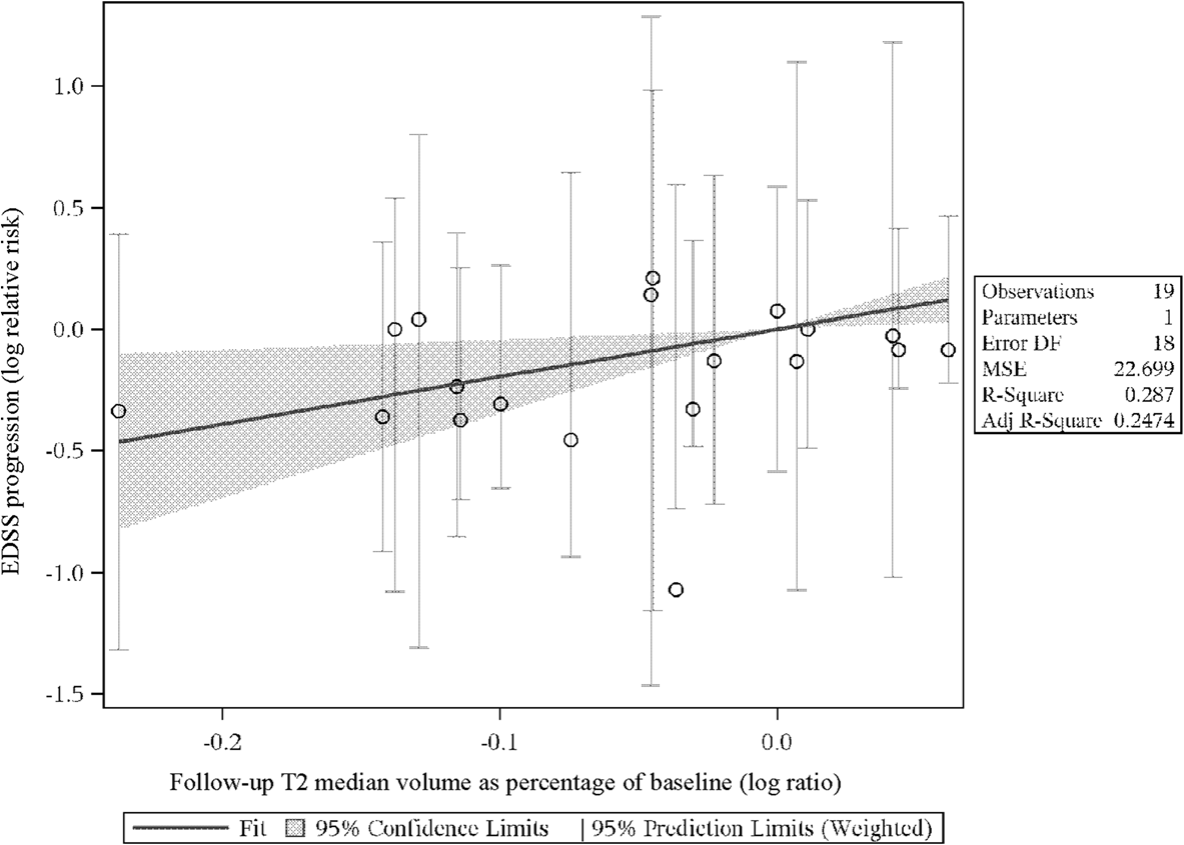
**Predicting log-relative risk of EDSS from log-rate ratio of median T2 lesion volume: RRMS, all DMTs.** EDSS: Expanded Disability Status Scale.

**Figure 4:**
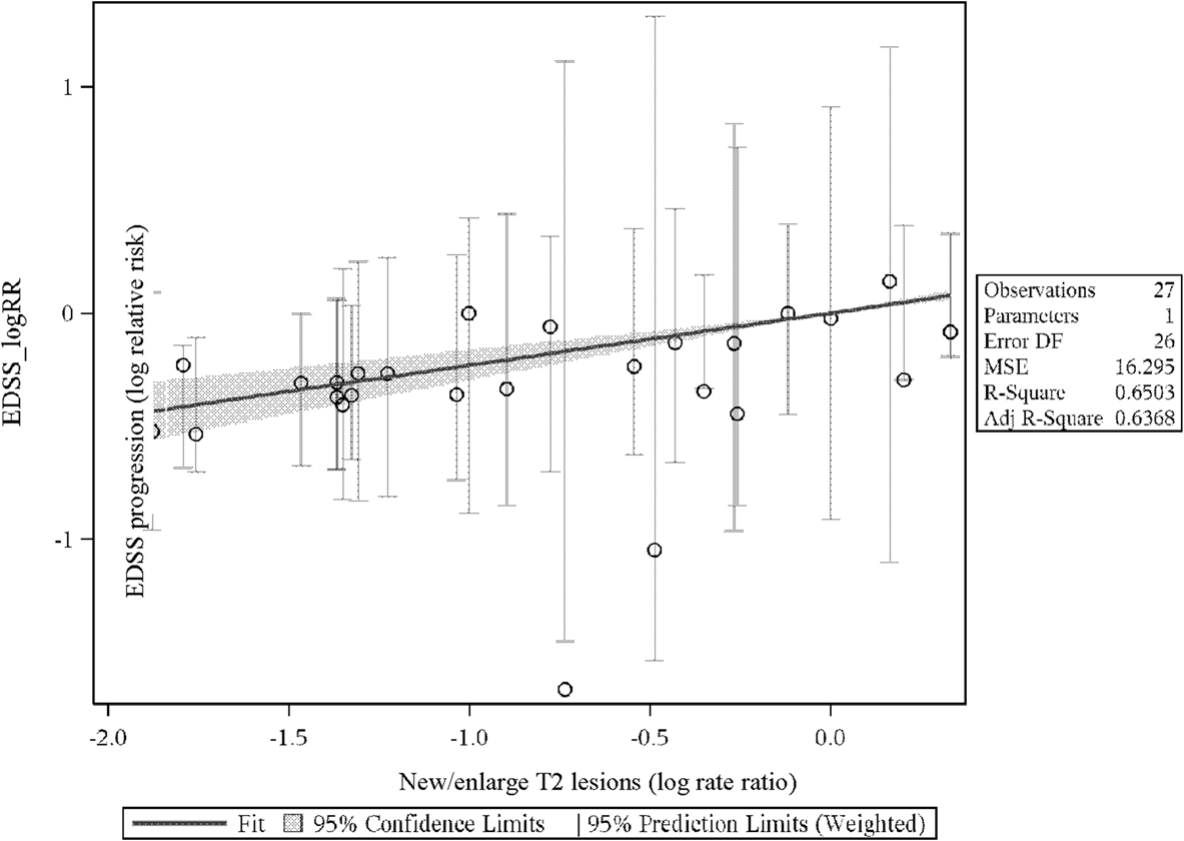
**Predicting log-relative risk of EDSS from log-rate ratio of active T2 lesions count.** EDSS: Expanded Disability Status Scale.

In the figures representing the analyses (Figures 2, 3 and 4), each slope suggests a predicted difference between any two treatments on an outcome (e.g., log-relative risk of EDSS progression) given a difference between those same treatments on a predictor (e.g., log-rate ratio of the ARRs). The trial data contributing to the analyses are provided in Tables 1 and 2. Evidence tables show the data for the predictors (i.e., ARR ratio, final T2 lesion volume ratio, ratio in number of new/enlarged T2 lesions) and the outcome (ratio of patients with confirmed disease progression).

**Table 1:** Study summaries in trials reporting relapse and disability progression

**Trial**	**Follow-up (months)**	**Comparison**	**Patients analyzed**	**ARR experimental arm**	**ARR control arm**	**Relapse rate ratio**	**EDSS progression experimental arm**	**EDSS Progression control arm**	**Disability risk ratio**
Comi G, et al., [17]	24	IFNB-1a 22 μg vs. Placebo	309	0.33	0.43	0.767	15.00	20.00	0.750
Hartung HP, et al. [24]	36	Mitoxantrone 12 mg vs. Placebo	124	0.35	1.02	0.343	8.00	22.00	0.364
Durelli L, et al. [18]	24	IFNB-1a 30 μg vs. IFNB-1b 250 μg	188	0.50	0.70	0.714	13.00	30.00	0.433
Rudick RA, et al. [38]	24	IFNB-1a plus Natalizumab vs. IFNB- 1a	1,171	0.34	0.75	0.453	23.00	29.00	0.793
Havrdova E, et al. [25]	24	IFNB-1a plus azathioprinethioprine vs. IFNB-1a	116	0.91	1.05	0.867	20.70	16.80	1.232
Havrdova E, et al. [25]	24	IFNB-1a plus azathioprine plus prednisone vs. IFNB-1a	120	0.73	1.05	0.695	17.50	16.80	1.042
Mikol DD, et al. [32]	22	IFNB-1a vs. Glatiramer acetate	764	0.29	0.30	0.967	8.70	11.70	0.744
Ravnborg M, et al. [37]	36	IFNB-1a plus Methylprednisolone vs. IFNB-1a	338	0.21	0.33	0.636	25.00	28.50	0.877
Sorensen P, et al. [39]	22	IFNB-1a plus Methylprednisolone vs. IFNB-1a	130	0.22	0.59	0.373	16.00	25.00	0.640
O’Connor P, et al. [34]	42	Glatiramer acetate vs. IFNB-1b 250 μg	1,158	0.34	0.36	0.944	20.50	22.30	0.919
O’Connor P, et al. [34]	42	Glatiramer acetate vs. IFNB-1b 500 μg	1,100	0.34	0.33	1.030	20.50	22.30	0.919
Gonsette RE, et al. [23]	24	IFNB-1b plus Inosine vs. IFNB-1b	157	0.50	0.38	1.316	17.78	18.18	0.978
Coles AJ, et al. [16]	36	Alemtuzumab vs. IFNB-1a	333	0.1	0.36	0.278	9.00	26.20	0.344
Kappos L, et al. [30]	24	Fingolimod 0.5 mg vs. Placebo	843	0.18	0.40	0.450	17.70	24.10	0.734
Kappos L, et al. [30]	24	Fingolimod 1.25 mg vs. Placebo	847	0.16	0.40	0.400	16.60	24.10	0.689
Polman CH, et al. [36]	24	Natalizumab vs. Placebo	942	0.23	0.73	0.315	17.00	29.00	0.586
Clanet M, et al. [14]	36	IFNB-1a 30 μg vs. IFNB-1a 60 μg	802	0.81	0.77	1.052	37.00	37.00	1.000
Ebers GC, et al. [9]	104	IFNB-1a 22 μg vs. Placebo	376	0.91	1.28	0.711	30.00	38.00	0.789
Ebers GC, et al. [9]	104	IFNB-1a 44 μg vs. Placebo	373	0.87	1.28	0.676	26.50	38.00	0.697
Johnson KP, et al. [27]	24	Glatiramer acetate vs. Placebo	251	0.59	0.84	0.702	21.60	24.60	0.878
Achiron A, et al. [11]	24	Immunoglobulin vs. Placebo	40	0.59	1.61	0.366	13.70	17.10	0.801
Baumhackl U, et al. [13]	24	Hydrolytic enzymes vs. Placebo	291	0.63	0.74	0.851	28.00	26.00	1.077
Fazekas F, et al. [20]	24	Immunoglobulin vs. Placebo	148	0.52	1.26	0.413	17.00	23.00	0.739
Millefiorini E, et al.[33]	24	Mitoxantrone vs. Placebo	51	0.445	1.31	0.340	7.00	37.00	0.189
Jacobs LD, et al. [26]	24	IFNB-1a vs. Placebo	172	0.61	0.90	0.678	21.10	33.30	0.633
Ebers GC, et al. [8]	36	IFNB-1b 1.6 MIU vs. Placebo	228	1.05	1.21	0.868	28.00	28.00	1.000
Ebers GC, et al. [8]	36	IFNB-1b 8 MIU vs. Placebo	227	0.84	1.21	0.694	20.00	28.00	0.714
van de Wyngaert FA, et al. [40]	36	Mitoxantrone vs. Methylprednisolone	28	0.26	1.00	0.260	21.00	21.00	1.000
Andersen O et al. [12]	36	IFNB-1a 22 μg vs. Placebo	364	0.25	0.27	0.926	41.00	38.00	1.079
Cohen JA, et al. [15]	24	IFNB-1a 60 μg vs. Placebo	379	0.20	0.30	0.667	28.50	33.70	0.846
Panitch H, et al. [35]	36	IFNB-1b 160 μg vs. Placebo	622	0.20	0.28	0.714	39.00	34.00	1.147
Panitch H, et al. [35]	36	IFNB-1b 250 μg vs. Placebo	625	0.16	0.28	0.571	32.00	34.00	0.941
Kappos L, et al. [28]	36	IFNB-1b vs. Placebo	718	0.44	0.64	0.688	38.90	49.70	0.783
Edan G, et al. [41]	36	Mitoxantrone 12 mg/m^2^ vs. IFNβ-1b 250 μg	109	0.39	0.71	0.549	9.10	25.90	0.351
Freedman MS, et al. [42]	24	MBP8298 vs. Placebo in DR2+ and/or DR4+ haplotypes	513	0.13	0.14	0.929	30.70	27.80	1.104
Freedman MS, et al. [42]	24	MBP8298 vs. Placebo in DR2- and/or DR4- haplotypes	99	0.08	0.20	0.400	28.30	35.80	0.791
Comi G, et al. [45]	24	Laquinimod 0.6 mg vs. Placebo	1,106	0.30	0.39	0.769	11.10	15.70	0.707
Cohen JA, et al. [43]	24	Alemtuzumab 12 mg vs. IFNβ-1a 44 μg	563	0.18	0.39	0.462	8.00	11.12	0.719
Coles AJ, et al. [44]	24	Alemtuzumab 12 mg vs. IFNβ-1a 44 μg	667	0.26	0.52	0.500	12.71	21.13	0.602
Fox RJ, et al. [46]	24	Dimethyl fumarate 240 mg BID vs. Placebo	480	0.22	0.40	0.550	13.00	17.00	0.765
Fox RJ, et al. [46]	24	Dimethyl fumarate 240 mg TID vs. Placebo	466	0.20	0.40	0.500	13.00	17.00	0.765
Fox RJ, et al. [46]	24	Glatiramer acetate vs. Placebo	471	0.29	0.40	0.725	16.00	17.00	0.941

ARR, annualized relapse rate; BID, twice daily; EDSS, Expanded Disability Status Scale; IFNB, Interferon-beta; μg, microgram; mg, milligram; mg/m2, milligrams per square meter of body surface; MIU, million international units; TID, three times daily.

**Table 2:** Study summaries in trials reporting MRI changes and disability progression

**Trial**	**Follow-up (months)**	**Comparison**	**Patients Analyzed**	**Change in T2 Lesion Volume Experimental Arm**	**Change in T2 Lesion Volume Control Arm**	**Final T2 Lesion Volume Ratio**	**New and/or Enlarged T2 Lesion Experimental Arm**	**New and or Enlarged T2 Lesion Control Arm**	**T2 Lesion Count Ratio**	**EDSS Progression Experimental Arm**	**EDSS Progression Control Arm**	**Disability Risk Ratio**
Kappos L, et al. [29]	24	IFNB-1b 250 μg vs. Placebo	468	-10.60	-5.00	0.941	3.70	8.50	0.435	12.00	20.00	0.600
Comi G, et al. [17]	24	IFNB-1a 22 μg vs. Placebo	300	-13.00	8.80	0.800	2.00	3.00	0.667	15.00	20.00	0.750
Hartung HP, et al. [24]	36	Mitoxantrone 12 mg vs. Placebo	124				0.29	1.94	0.149	8.00	22.00	0.364
Rudick RA, et al. [38]	24	IFNB-1a plus Natalizumab vs. IFNB 1a	1171				0.90	5.40	0.167	23.00	29.00	0.793
Havrdova E, et al. [25]	24	IFNB-1a plus Azathioprinethioprine vs. IFNB-1a	88	24.60	30.30	0.956				20.70	16.80	1.232
Havrdova E, et al. [25]	24	IFNB-1a plus azathioprine plus prednisone vs. IFNB-1a	93	14.50	30.30	0.879				17.50	16.80	1.042
Mikol DD, et al. [32]	22	Glatiramer acetate vs. IFNB-1a	585				0.82	0.67	1.224	8.70	11.70	0.744
Ravnborg M, et al. [37]	36	IFNB-1a plus Methylprednisolone vs. IFNB-1a	220	-1.06	1.21	0.978	5.20	8.00	0.650	25.00	28.50	0.877
Sorensen PS, et al. [39]	22	IFNB-1a plus Methylprednisolone vs. IFNB-1a	110				2.70	3.50	0.771	16.00	25.00	0.640
O’Connor P, et al. [34]	42	Glatiramer acetate vs. IFNB-1b 250 μg	913	17.00	10.00	1.064	4.60	3.30	1.394	20.50	22.30	0.919
O’Connor P, et al. [34]	42	Glatiramer acetate vs. IFNB-1b 500 μg	971	17.00	12.00	1.045	4.60	3.30	1.394	20.50	22.30	0.919
Freedman MS, et al. [21]	36	Placebo/IFNB-1a 44 μg vs. Placebo/IFNB-1a 22 μg	53	-3.40	1.10	0.955	2.00	1.70	1.176	46.00	40.00	1.150
Freedman MS, et al. [21]	36	Placebo/IFNB-1a 44 μg vs. IFNB-1a 22 μg	83	5.40	1.10	1.043	1.70	1.70	1.000	39.00	40.00	0.975
Freedman MS, et al. [21]	36	Placebo/IFNB-1a 44 μg vs. IFNB-1a 22 μg	85	1.80	1.10	1.007	1.30	1.70	0.765	35.00	40.00	0.875
Coles AJ, et al. [16]	36	Alemtuzumab vs. IFNB-1a	227	-16.40	-13.30	0.964				9.00	26.20	0.344
Kappos L, et al. [30]	24	Fingolimod 0.5 mg vs. Placebo	537	-1.69	8.61	0.905	2.50	9.80	0.255	17.70	24.10	0.734
Kappos L, et al. [30]	24	Fingolimod 1.25 mg vs. Placebo	512	-3.10	8.61	0.892	2.50	9.80	0.255	16.60	24.10	0.689
Polman CH, et al. [36]	24	Natalizumab vs. Placebo	942				1.90	11.00	0.173	17.00	29.00	0.586
Clanet M et al. [14]	36	IFNB-1a 60 μg vs. IFNB-1a 30 μg	386	-0.20	-1.29	1.011	8.00	9.00	0.889	37.00	37.00	1.000
Ebers GC, et al. [9]		IFNB-1a 22 μg vs. Placebo	279	-1.20	10.90	0.891	9.00	15.50	0.581	30.00	38.00	0.789
Ebers GC, et al. [9]		IFNB-1a 44 μg vs. Placebo	281	-3.80	10.90	0.867	5.50	15.50	0.355	26.50	38.00	0.697
Baumhackl U, et al. [13]	24	Hydrolytic enzymes vs. Placebo	291	-1.00	-1.00	1.000				28.00	26.00	1.077
Millefiorini E, et al. [33]	24	Mitoxantrone vs. Placebo	42				3.50	7.30	0.479	7.00	37.00	0.189
Giovannoni G, et al. [22]	22	Cladribine 5.25 mg/kg bw vs. Placebo	674				0.33	1.43	0.231	15.10	20.60	0.733
Giovannoni G, et al. [22]	22	Cladribine 3.5 mg/kg bw vs. Placebo	651				0.38	1.43	0.266	14.30	20.60	0.694
Jacobs LD, et al. [26]	24	IFNB-1a vs. Placebo	164	-13.20	-6.50	0.928				21.10	33.30	0.633
Ebers GC, et al. [8]	36	IFNB-1b 1.6 MIU vs. Placebo	164	0.20	15.00	0.871	1.80	4.90	0.367	28.00	28.00	1.000
Ebers GC, et al. [8]	36	IFNB-1b 8 MIU vs. Placebo	167	-9.30	15.00	0.789	2.00	4.90	0.408	20.00	28.00	0.714
Panitch H, et al. [35]	36	IFNB-1b 160 μg vs. Placebo	81	0.80	10.90	0.909				39.00	34.00	1.147
Panitch H, et al. [35]	36	IFNB-1b 250 μg vs. Placebo	81	0.40	10.90	0.905				32.00	34.00	0.941
Edan G, et al. [41]	36	Mitoxantrone 12 mg/m^2^ vs. IFNβ-1b 250 μg	109				2.15	3.50	0.614	9.10	25.90	0.351
Freedman MS, et al. [42]	24	MBP8298 vs. Placebo in DR2+ and/or DR4+ haplotypes	513	0.90	6.20	0.95	3.90	3.30	1.182	30.70	27.80	1.104
Freedman MS, et al. [42]	24	MBP8298 vs. Placebo in DR2- and/or DR4- haplotypes	99	16.10	0.70	1.15	3.20	3.30	0.970	28.30	35.80	0.791
Comi G, et al. [45]	24	Laquinimod 0.6 mg vs. Placebo	1,106				5.03	7.14	0.704	11.10	15.70	0.707
Cohen JA, et al. [43]	24	Alemtuzumab 12 mg vs. IFNβ-1a 44 μg	563	-9.30	-6.50	0.97				8.00	11.12	0.719
Coles AJ, et al. [44]	24	Alemtuzumab 12 mg vs. IFNβ-1a 44 μg	667	-1.27	-1.23	1.00				12.71	21.13	0.602
Gold R, et al. [47]	24	Dimethyl fumarate 240 mg BID vs. Placebo	818				2.60	17.00	0.153	16.00	27.00	0.593
Gold R, et al. [47]	24	Dimethyl fumarate 240 mg TID vs. Placebo	824				4.40	17.00	0.259	18.00	27.00	0.667
Fox RJ, et al. [46]	24	Dimethyl fumarate 240 mg BID vs. Placebo	480				5.10	17.40	0.293	13.00	17.00	0.765
Fox RJ, et al. [46]	24	Dimethyl fumarate 240 mg TID vs. Placebo	466				4.70	17.40	0.270	13.00	17.00	0.765
Fox RJ, et al. [46]	24	Glatiramer acetate vs. Placebo	471				8.00	17.40	0.460	16.00	17.00	0.941

BID, twice daily; EDSS, Expanded Disability Status Scale; IFNB, Interferon-beta; μg, microgram; mg, milligram; mg/m2, milligrams per square meter of body surface; mg/kg bw, milligram per kilogram of body weight; MIU, million international units; TID, three times daily.

### Predicting disability progression from relapse

The relationships between the log-ARR and log-relative-risk of EDSS progression were statistically significant across all analyses (*p* < 0.001 for RRMS-all DMTs and RRMS-approved DMTs, *p* = 0.027 for SPMS; see Table 3). The strength of the predictive power of log-ARR to predict EDSS progression did not vary substantively across analyses (slopes of 0.53, 0.50, and 0.40, respectively). Figure 2 plots the relationship between the log-ARR and log-relative risk of EDSS progression for all RRMS studies.

**Table 3:** Results of the regression analysis

**Model**	**Predictor**	**Slope**	***p*-value**	**Lower 95% CI**	**Upper 95% CI**	**Adjusted R**^**2**^
RRMS, All DMTs	Annual relapse ratio log-ratio	0.531	< 0.001	0.425	9.637	0.750
RRMS, Approved DMTs	Annual relapse ratio log-ratio	0.500	< 0.001	0.338	0.663	0.670
SPMS and SPMS Mixed	Annual relapse ratio log-ratio	0.397	0.027	0.060	0.735	0.414
RRMS, All DMTs	T2 median volume log-ratio	1.953	0.015	0.428	3.477	0.247
RRMS, Approved DMTs	T2 median volume log-ratio	1.407	0.018	0.281	2.533	0.3305
RRMS, Not Approved	T2 median volume log-ratio	3.978	0.151	-2.055	10.011	0.238
SPMS and SPMS Mixed	T2 median volume log-ratio	NA	NA	NA	NA	NA
RRMS, All DMTs	T2 lesion count log-ratio	0.138	< 0.001	0.079	0.196	0.706
RRMS, Approved DMTs	T2 lesion count log-ratio	0.162	0.025	0.027	0.298	0.467
SPMS and SPMS Mixed	T2 lesion count log-ratio	NA	NA	NA	NA	NA

DMT, disease-modifying treatment; RRMS, relapsing-remitting multiple sclerosis; SPMS, secondary progressive multiple sclerosis; NA, not available.

Each point in the plot represents a study comparison for these two effects. For instance, the most rightward point is from Gonsette et al. [23], which found almost no difference in EDSS progression between the two arms (approximately 18% for both) but a substantive difference in relapse rate (0.50 vs. 0.38, for a log-rate-ratio of 0.27). Each study with two arms (one treatment comparison, e.g., treatment A vs. treatment B) with sufficient data contributed one data point to the analysis; studies with three arms (two treatment comparisons, e.g., A vs. B and A vs. C) contributed two data points.

Any given slope can be interpreted by determining what difference between treatments in EDSS progression one would expect given a realistic difference in ARR ratio. A realistic difference in the ARR ratio can be operationalized as the median ARRR across all studies. In this data, the median ratio of the mean relapse rate in the active to the control group was 0.70. For instance, in Johnson et al. [27], the ARR for glatiramer acetate (GA) was 0.59, while it was 0.84 for placebo (relative risk [RR] = 0.70).

As ln(0.70) = -0.353, the predicted log-relative-risk of EDSS progression in studies like Johnson et al. is:

$$\ln\left(RR_{\text{EDSS}}\right)=\text{Slope}\;*\;\ln\left(\text{ARRR}\right),$$

Or

$$\ln\left(RR_{\text{EDSS}}\right)=0.53\left(-0.353\right),=-0.187.$$

As exp(-0.187) = 0.83, we can predict that the relative risk of EDSS progression in studies like Johnson et al. (i.e. with an ARRR of 0.70) will be 0.83. In Johnson et al., the relative risk of EDSS progression was similar to this value: it was 0.88 (21.6% progression in GA, 24.6% progression on placebo).

### Predicting disability progression from lesion volume

The slope for predicting log-relative-risk of EDSS progression from the log-ratio of median follow-up in T2 volume (expressed as a percentage of baseline volume) was statistically significant for the RRMS subset of studies (1.95, 95% CI [0.43, 3.48], *p* = 0.015) and marginally statistically significant for the subset of RRMS studies on approved DMTs (1.09, 95% CI [-0.09, 2.27], *p* = 0.067). There were insufficient data on T2 volume to conduct analyses on SPMS patients; only one study [35] had such data. Figure 3 plots the relationship between the log-rate ratio of median T2 lesion volume and log-relative risk of EDSS progression for all RRMS studies.

Following the example above, the median ratio of follow-up T2 lesion volume was 0.96. For instance, in Coles et al. 2008,[16] the median T2 lesion volume dropped by 16.4% on alemtuzumab and dropped 13.3% on placebo. This led to a ratio of 0.96 for the follow-up values (0.836/0.867) and a log-ratio of -0.036.

The predicted difference between treatments in EDSS progression in studies like Coles 2008 [16] is:

$$\ln\left({\mathrm{RR}}_{\text{EDSS}}\right)=\text{Slope}*\ln\left(\text{ratio}\;\mathrm{of}\;\text{median}\;T2\;\text{volume}\right),$$

Or

$$\ln\left({\mathrm{RR}}_{\text{EDSS}}\right)=1.95*\left(-0.036\right)=-0.070$$

As exp(-0.070) = 0.93, we predict that the relative risk of EDSS progression in studies like Coles et al. 2008 will be 0.93. The actual ratio in Coles et al. 2008 was much lower: 0.34. This is perhaps unsurprising, given the wide confidence interval for the slope (0.43–3.48); while we see a significant relationship between median T2 percentage volume and EDSS progression, it is not one that is precisely estimated.

Given the large difference in estimated slope between RRMS, approved DMTs, and RRMS overall, sensitivity analyses were conducted to explore the difference. A separate analysis was conducted limited to studies for which the active treatment has not been approved. The relationship between T2 lesion volume difference and the EDSS relative risk was much stronger in the RRMS studies lacking an approved DMT (slope = 3.98, 95% CI: –2.06–10.01) but was not statistically significant (*p* = 0.15). The high slope but wide confidence interval suggests that in these studies, T2 lesion volume changes much less, relative to change in EDSS progression, than in studies with approved DMTs. Specifically, there are sometimes small differences in lesion volume even though there are large differences in the percentage of patients with EDSS progression. This may be due to a different mechanism of action, but also may simply be an artifact of sampling error, given the wide confidence interval. If this signal is not an artifact, it may suggest that newer and/or less conventional therapies can affect lesion volume very little relative to their effect on short-term disease progression.

### Prediction of EDSS progression using (log)-ratio of mean number of active T2 lesions (RRMS, all DMTs, RRMS, approved DMTs)

The slopes for the prediction of the log-relative-risk of EDSS progression from the log-ratio of T2 lesions (generally defined as “new or enlarging” T2 lesions) were statistically significant (0.23 and 0.21 respectively, *p* < 0.001) for both the set of all RRMS studies and the set restricted to studies comparing approved DMTs. There were insufficient data on T2 lesion counts to conduct analyses on SPMS patients.

Following methods used previously, the median ratio of follow-up T2 lesion volume was 0.48. For instance, in Millefiorini et al. [33] the mean number of active T2 lesions was 7.3 in the placebo group and 3.5 in the mitoxantrone group (3.5/7.3 = 0.48, log-ratio = -0.74).

The predicted difference between treatments in EDSS progression in studies like Millefiorini et al. [33] is:

$$\ln\left({\mathrm{RR}}_{\text{EDSS}}\right)=0.23*\left(-0.74\right)=-0.17$$

As exp(-0.17) = 0.84, we predict that the relative risk of EDSS progression in studies like Millefiorini et al. will be 0.84. The actual ratio in Millefiorini et al. was much lower: 0.19; this study was an outlier, with only 7% of patients progressing on active treatment, while 37% progressed on placebo. However, the study had a very small sample size of the study (42 patients with MRI data), making it unsurprising that the predicted value is not close to the study value. Figure 4 plots the relationship between the log-rate ratio of active T2 lesion count and log-relative risk of EDSS progression for all RRMS studies.

## Discussion

This literature review and analysis demonstrates significant links between the therapeutic impact of DMTs on EDSS progression and changes on surrogate markers of disability, namely annualized relapse rates and T2 lesion counts and volumes. This analysis improves upon the Sormani et al. [6] analysis by including DMT studies of SPMS, including RRMS studies published through June of 2013, using revised data from the O’Connor et al. [34] study, examining T2 lesion volume in addition to counts of new and enlarging lesions, and exclusion of an intercept in the regression analyses. Even though our methodological approach was different, we found substantively similar results.

While conclusions cannot be drawn regarding the impact of treating relapse on long-term disability progression, this analysis confirms that these relationships exist over the short term (approximately two years) after treatment initiation. We found a substantive clinically and statistically significant link between concurrent treatment effects in relapse rate and in EDSS. Specifically, studies with lower relative differences in relapse rate had lower relative differences in EDSS progression: the stronger the treatment effect on reducing ARR, the stronger the effect found for reducing disease progression. There was no conclusive evidence that the relationships had different strengths between approved and non-approved DMTs in RRMS patients or between types of MS (RRMS and SPMS) patients, but the sparseness of data and the presence of outliers in some analyses prohibit strong generalizations. It remains possible that differences in the mechanism of action between newer DMTs and older DMTs may result in different relapse/EDSS relationships. However, despite our review including more than twice as many studies compared with Sormani et al. (40 vs. 19), several of which evaluated more recently approved DMTs such as dimethyl fumarate, laquinimod, and fingolimod, the findings in the two analyses are similar.

Significant association between T2 lesion measures and EDSS measures were also found. Because not all studies contributed data on all predictors and outcomes of interest (e.g., not all studies reporting median T2 volume change also reported the number of active T2 lesions, and vice versa), it is difficult to make conclusions with regard to what predictors and outcome pairs have the strongest relationships. However, the values of adjusted-R^2^ were higher and the relative confidence-interval widths were narrower when the log-ratio of active T2 lesion counts was the predictor of EDSS progression. It may be that the wide variation present in T2 lesion volume blurs its relationship with disability progression. Our results suggest that counting new and/or enlarged lesions is a better way to predict concurrent disability progression than lesion volume, but more research is needed to confirm this supposition.

Since this analysis used only aggregated summary data from published studies, we cannot necessarily assume that any statistical association observed between group-level variables may be translated to patient-level associations. Therefore, our findings cannot be used to predict any outcome at the patient-level. In addition, unlike some surrogate endpoint analyses [7], the relationships in the current investigation are concurrent. Thus, even though relationships were found between, for example, ARR ratios and relative risk of EDSS progression, it does not guarantee that early differences in relapse rate can predict later differences in EDSS progression.

## Conclusions

While it remains possible that early relapses are concomitant with, rather than causative of, disease progression, treatment differences in relapse reduction are significantly related to differences in disease progression over the first two years of treatment. Similarly, treatment differences in T2 lesion measures are also predictive of treatment differences in relapse rates over the first two years.

## Competing interests

Kyle Fahrbach, Rachel Huelin, and Amber Martin are employees of Evidera, which received funding from Novartis Pharmaceuticals Corporation to conduct the study on which this manuscript is based. Homa Dastani was an employee of Novartis at the time the work was completed but is now employed by Bristol-Myers Squibb Company. Manoj Malhotra was an employee of Novartis at the time the work was completed but is now employed by Questcor Pharmaceuticals. Edward Kim is an employee of Novartis. Stephen Rao received honoraria from Novartis for consulting on this project.

## Authors’ contributions

All authors were involved in developing the design for the study. KF, RH and AM carried out the design and interpretation of results. KF conducted all analyses. All authors have read and approved the manuscript.

## Pre-publication history

The pre-publication history for this paper can be accessed here:

http://www.biomedcentral.com/1471-2377/13/180/prepub
